# Identification of a Key Amino Acid of LuxS Involved in AI-2 Production in *Campylobacter jejuni*

**DOI:** 10.1371/journal.pone.0015876

**Published:** 2011-01-11

**Authors:** Paul Plummer, Jinge Zhu, Masato Akiba, Dehua Pei, Qijing Zhang

**Affiliations:** 1 Department of Veterinary Microbiology and Preventative Medicine, College of Veterinary Medicine, Iowa State University, Ames, Iowa, United States of America; 2 Department of Veterinary Diagnostics and Production Animal Medicine, College of Veterinary Medicine, Iowa State University, Ames, Iowa, United States of America; 3 Department of Chemistry, The Ohio State University, Columbus, Ohio, United States of America; University of Hyderabad, India

## Abstract

Autoinducer-2 (AI-2) mediated quorum sensing has been associated with the expression of virulence factors in a number of pathogenic organisms and has been demonstrated to play a role in motility and cytolethal distending toxin (cdt) production in *Campylobacter jejuni*. We have initiated the work to determine the molecular basis of AI-2 synthesis and the biological functions of quorum sensing in *C. jejuni*. In this work, two naturally occurring variants of *C. jejuni* 81116 were identified, one producing high-levels of AI-2 while the other is defective in AI-2 synthesis. Sequence analysis revealed a G92D mutation in the *luxS* gene of the defective variant. Complementation of the AI-2^−^ variant with a plasmid encoded copy of the wild-type *luxS* gene or reversion of the G92D mutation by site-directed mutagenesis fully restored AI-2 production by the variant. These results indicate that the G92D mutation alone is responsible for the loss of AI-2 activity in *C. jejuni.* Kinetic analyses showed that the G92D LuxS has a ∼100-fold reduced catalytic activity relative to the wild-type enzyme. Findings from this study identify a previously undescribed amino acid that is essential for AI-2 production by LuxS and provide a unique isogenic pair of naturally occurring variants for us to dissect the functions of AI-2 mediated quorum sensing in *Campylobacter*.

## Introduction

The role of quorum sensing in the adaptation and colonization of bacterial pathogens has been of increasing interest over the last decade. A growing body of evidence suggests that the direct sensing of threshold levels of various quorum sensing compounds is associated with changes in gene regulation resulting in altered phenotypic expression of virulence factors. [1], [2], [3]. Examples of such changes include regulation of adherence, motility, toxin production and expression of type three secretion systems in a variety of bacterial species. [4], [5], [6], [7], [8], [9], [10]. While the quorum sensing mechanisms of Gram-positive bacteria are often associated with the production and sensing of modified peptide signals, the autoinducers of Gram-negative bacteria are more commonly acyl-homoserine lactones[11], [12]. Another form of quorum-sensing, mediated by autoinducer-2, has been described as a highly conserved inter-species mechanism of communication with genetic conservation over a large number of both Gram-positive and Gram-negative bacteria [12], [13]. Production of AI-2 requires the activity of a conserved LuxS protein, a *S*-ribosylhomocysteinase that converts *S*-ribosylhomocysteine into homocysteine and 4,5-dihydroxy-2,3-pentanedione (DPD) [14]. Under physiological conditions, DPD spontaneously cyclizes to form a mixture of several furanones which are collectively referred to as AI-2.

LuxS is a Fe^2+^ metalloenzyme whose catalytic mechanism has been extensively studied and well defined [14], [15], [16], [17]. Two universally conserved residues, Cys-84 and Glu-57 (amino acid numbering in *E. coli* LuxS), have been shown to act as critical general acids/bases during catalysis. In addition, several other active-site residues including Ser-6, His-11 and Arg-39 are also important for full activity [14], [17].

*Campylobacter jejuni* is a leading cause of foodborne bacterial enteritis in the United States and many developed countries [18], [19], [20]. Foodborne cases are most commonly associated with the consumption of contaminated poultry products, milk or drinking water [20]. Although the majority of cases result in relatively mild self-resolving enteritis, severe infections require treatment with antimicrobial compounds [21]. The flouroquinolone and macrolide classes of antimicrobials represent the most commonly prescribed drugs used in severe human infections [22]. An increasing prevalence of antimicrobial resistant *Campylobacter* isolates has resulted in a renewed effort to identify novel means of control for this bacterial pathogen [22], [23]. Results in other bacterial species have suggested that the quorum sensing mechanisms may represent a viable target for bacterial control [24], [25], [26], [27], [28], [29], however there are significant knowledge gaps in our understanding of the quorum sensing mechanisms of *C. jejuni.*

In order to determine the viability of utilizing quorum sensing mechanisms of *Campylobacter* as a therapeutic target we must first expand our understanding of both the molecular mechanisms responsible for autoinducer production and their role in virulence. Currently, evidence suggests that AI-2 mediated quorum sensing may play a role in motility, *in vitro* bacterial agglutination, and the production of cytolethal distending toxin by *Campylobacter jejuni* [5], [7], [8], [30]. This paper details the identification and description of a naturally occurring LuxS mutant strain of *Campylobacter jejuni* and provides new insights into the molecular mechanisms of LuxS production and function in this organism.

## Materials and Methods

### Strains and Culture Media/Conditions

The parent strain used in this study was *Campylobacter jejuni* 81116 (NCTC11828), an isolate obtained from a human enteritis outbreak in a boarding school located in the United Kingdom in 1983 [31]. The 81116AI2- variant is the AI-2 deficient clone of this strain maintained and passaged in the author's laboratory. The 81116AI2+ strain is the AI-2 competent clone obtained from an independent laboratory. Other strains utilized in this study are detailed in Table 1. All strains are maintained in 20% glycerol stock at −80°C. *C. jejuni* strains were passaged on Mueller-Hinton agar (without blood) or broth in a microaerophilic environment generated by the use of compressed gas (5% O_2_, 10% CO_2_, 85% N_2_). Routine cultures were incubated at 42°C. *Campylobacter jejuni* strains with antimicrobial resistance markers were grown on kanamycin (30 µg/ml) or chloramphenicol (4 µg/ml) when appropriate. All *Escherchia coli* strains used for genetic manipulation were grown in Luria-Bertani (LB) broth or agar and incubated in a normal atmosphere at 37°C. When appropriate kanamycin (40 µg/ml), chloramphenicol (20 µg/ml), or ampicillin (30 µg/ml) was added to the culture media. *Vibrio harveyi* strains were grown in autoinducer broth (AB Broth) as described previously [32].

**Table 1:** Bacterial strains and plasmids used in this study.

Bacterial Strains	Description	Reference
81116AI2-	A variant of *C. jejuni* 81116 negative with AI-2	This study
LuxS-	81116AI2- LuxS insertional mutant	This Study
AI2-(pLuxS)	81116AI2- with plasmid encoded 11168 LuxS	This Study
AI2-(p256G)	81116AI2- with plasmid encoded site directed revertant of81116AI2- LuxS ORF	This Study
81116AI2+	*C. jejuni* 81116 positive with AI-2	[31]
11168	C. jejuni NCTC 11168	[48]
11168ΔluxS	11168 mutant with *kan* ^R^ inserted into luxS	This Study
BB170	AI-2 reporter strain; luxN::Tn5	[49]
BB152	AI-1-, AI-2+; luxM::Tn5	[32]
**Plasmids**		
pRY111	*E. coli-Campylobacter* shuttle plasmid, Kan^R^	[35]
puc256G	puc19 plasmid containing site directed revertant	This Study
pRY256G	pRY111 backbone with site directed revertant LuxS	This Study

#### Insertional Mutation of the *LuxS* gene of *C. jejuni*

An isogenic *luxS* mutant of *C. jejuni* NCTC 11168 was constructed by insertional mutagenesis. Primers luxSF4 and luxSR4 (Table 2) were used to amplify a 445 bp *luxS* fragment with a unique HindIII site in the middle region of the fragment. The PCR fragment was cloned into the pGEM-T Easy vector (Promega), resulting in the construction of pLUXS. Primers kanF and kanR (Table 2) were used to amplify the *kan* gene encoding kanamycin resistance from KAN-2 Transposon using the *PfuTurbo* DNA polymerase (Stratagene). After the HindIII digestion, the *kan* PCR product was ligated to the HindIII digested pLUXS to obtain plasmid construct pLUXSK, which was then transformed into *E. coli* JM109. pLUXSK, which serve as a suicide vector in *Campylobacter*, was introduced into *C. jejuni* NCTC 11168 using an electroporator (Gene Pulser Xcell System; Bio-Rad Laboratories). Transformants were selected on MH agar containing kanamycin 50 µg/ml. PCR analysis of the *luxS* mutant confirmed that the *kan* gene was inserted into the *luxS* gene in the same orientation as the *luxS* gene. Natural transformation was used to move this gene insertion into additional *C. jejuni* strains as previously described [33].

**Table 2:** Primers used in this study.

Primers	Sequence	Target Gene
Cj1198F1	GTTGGATCCCCATTATTAGACAGCTTTAAAGT	*luxS*
Cj1198R3	TAATCTGCAGTTTAAGCATTCTCGAGTTTT	*luxS*
LuxSF4	ACCTAAGGGTGATGATATTA	*luxS*
LuxSR4	GCTTAATCATTTAAAAAATAA	*luxS*
KanF	TTAAGCTTGCATCGATGAATTGTGTCTCAAAA	Kn^R^
KanR	TTAAGCTTGGTGGACCAGTTGGTGATTT	Kn^R^
luxSF7	TAGTCTAGATCATTTAAAACAGGCAAAGC	*luxS*
luxSR9	GTCCTGCAGCACATCTCGCACATCAGTT	*luxS*
luxS256G	P-GTTGTCGCACGGGTTTTTATATGAG	*luxS*

#### *Vibrio harveyi* autoinducer-2 bioassay

Autoinducer-2 (AI-2) levels of cell-free spent culture media were measured using the *Vibrio harveyi* bioluminescence assay as previously described [34]. Briefly, the cell-free spent media was prepared by centrifugation of the sample at 10,000×*g* for 5 minutes followed by filter sterilization through a 0.25 µm syringe filter. Cell-free supernatant was immediately frozen at −80°C until being subjected to AI-2 measurement. A 10 µL aliquot of each sample was used in the bioluminescence assay and the relative light units of bioluminescence were measured using a Turner Biosystems Reporter Luminometer. Cell free supernatant of *Vibrio harveyi* strain BB152 was used as a positive control and LB and MH broth media were both used as negative controls. All measurements were reported at the 3 hour incubation period when the difference between negative controls and positive controls reached maximal levels. Results reported are the average of three independent assays.

### Complementation of *LuxS* deficient variant *in trans*

Complementation of AI-2 phenotype of the Cj81116AI2- variant was performed using a plasmid-carried gene. Briefly, the *luxS* open reading frame and 500 bp of the upstream region of non-coding sequence of Cj81116AI2+ were cloned into the pRY111 vector [35]. The vector was conjugated into 81116AI2- as described below and selected using MH media plus chloramphenicol (4 µg/mL).

#### Site-directed mutagenesis

Site-directed mutagenesis of the single nucleotide substitution found in *C. jejuni* strain 81116AI2- was performed in order to revert the substituted aspartic acid residue to the wild-type glycine found in the AI-2 competent 81116AI2+ strain. Site-directed mutagenesis was performed using the Transformer Site-Directed Mutagenesis Kit (Clontech) according to the manufacturer's recommendations. Briefly, the full *luxS* ORF and approximately 500 bp of the upstream non-coding promoter region of strain 81116AI2- was amplified using the luxSF7 (including a *Pst* I site) and luxSR9 (including a *Xba* I site) primers. The PCR products and the puc19 plasmid were each individually subjected to restriction enzyme digestion with *Pst* I and *Xba* I. Following digestion the samples were purified using a Qiagen PCR Clean-Up column and ligated using T_4_ ligase (Promega). The plasmid was transformed into chemically competent DH5α cells (Invitrogen) and selected using LB media containing 100 µg/ml ampicillin, X-Gal and IPTG. As single clone was selected and DNA sequencing confirmed the plasmid contained the appropriate insert. Site-directed mutagenesis was performed using the 5′-phosphorylated control selection primer provided in the kit and a 5′-phosphorylated mutagenesis primer designed in this study and called luxS256G. The mutagenesis primer was designed to replace the adenine at nucleotide 256 of the *C. jejuni luxS* ORF with a guanine. Selection of desired mutants was performed as described by the manufacturer using *Nde* I restriction enzyme digestion. DNA sequencing was utilized to verify the isolation of puc256G, the puc19 plasmid containing the 81116AI2- *luxS* ORF with the A256G nucleotide mutation. PCR amplification of the mutated *luxS* ORF and promoter region in the puc256G plasmid was performed using the luxSF7 and luxSR9 primers used above, digested with *Pst* I and *Xba* I and ligated into a similarly digested pRY111 *C. jejuni* shuttle vector to yield the pRY256G plasmid. To transfer the pRY256G shuttle vector into the *C. jejuni* 81116AI2- strain, conjugation was performed as previously described [36].

#### Production of histidine-tagged recombinant LuxS protein

Six-histidine-tagged recombinant proteins were produced in *E. coli* JM109. The *luxS* ORF of both *C jejuni* 81116AI2- and *C. jejuni* 81116AI2+ were cloned in frame into the multicloning site of the pQE30 expression vector (Qiagen). The ORF was amplified by PCR using primers Cj1198F1 (including a *Bam* HI site) and Cj1198R3 (including a *Pst* I site), digested with *Bam* HI and *Pst* I, and ligated into a BamHI/PstI digested pQE30 plasmid. The authenticity of the plasmid was confirmed by sequencing the entire *luxS* gene. Recombinant proteins were produced following the manufacturer's recommendations for the pQE30 expression system. The eluted fraction was diluted to a total volume of 2.5 mL with 10 mM phosphate buffer, desalted using a PD-10 desalting column (Amersham) and concentrated using a Millipore centrifugal concentration column with a 10,000 MW size exclusion (Millipore). The concentration of the recombinant protein was determined using a standard BCA protein assay kit (Pierce). The protein was diluted to 1 mg/mL and flash frozen at −80°C in small aliquots. Cobalt-substituted LuxS was prepared as previously described [16]. Briefly, cells were grown in minimal media containing Co^2+^. At the time of induction, cobalt chloride was added to a final concentration of 10 µM, along with IPTG. The cells (from x l) were collected by centrifugation and resuspended in 4 ml of a lysis buffer (20 mM Tris, pH 8.0, 0.5 M NaCl, 5 mM imidazole, 1% Triton X-100, 0.5% protamine sulfate and 70 µg/mL chicken egg lysozyme). The sample was incubated at 4°C for 20 min and centrifuged. The supernatant was mixed with 1.5 mL of Talon Cobalt Purification resin (Clontech) and the mixture was transferred into a 5-mL column (Qiagen). The column was washed with 2 column volumes of washing buffer (20 mM Tris (pH 8.0), 0.5 M NaCl, and 5 mM imidazole) and the bound protein was eluted with 5 mL of elution buffer (20 mM Tris (pH 8.0), 0.5 M NaCl, and 60 mM imidazole). The eluted fraction was concentrated using a Centricon 10 (10,000 MW cutoff) to about half their volume and the protein was quantitated using a BCA Protein Assay Kit (Pierce). Glycerol was added to a final concentration of 20% and the proteins were flash frozen at −80°C.

#### Circular dichroism of recombinant proteins

Circular dichroism analysis of the recombinant LuxS proteins from 81116AI2- and 81116AI2+ were performed to compare the secondary structure of the proteins. The proteins were tested in a 10 mM phosphate buffer using a Jasco J-710 Spectropolarimeter. Measurements were recorded at a 0.2 nm resolution from 190 nm to 260 nm with a sensitivity of 20 mdeg. The assay was performed at room temperature.

#### *In vitro* synthesis of *S-*ribosylhomocysteine

*S*-ribosylhomocysteine was synthesized as previously described with slight modification [37]. A 10 mg/mL solution of *S*-adenosylhomocysteine (Sigma) was dissolved in 0.1 M HCl, boiled for 90 min and neutralized with an equal volume of 0.1 M NaOH [38]. ESI positive and negative mode mass spectroscopy was used to verify the disappearance of the SAH peak at 385 m/z and the presence of the expected 266 m/z SRH peak. This method has previously been demonstrated to provide a relatively pure SRH product [37]. Furthermore, incubation of the synthesized product with recombinant LuxS wildtype enzyme clearly demonstrated AI-2 synthesis while incubation of the recombinant enzyme with the SAH failed to produce measureable AI-2 or homocysteine.

#### *In vitro* AI-2 synthesis reaction

The ability of the recombinant LuxS proteins to synthesize biologically active AI-2 was assessed essentially as previously described [37]. Briefly, 100 µL of recombinant protein (1 mg/mL) was added to 300 µL of synthesized SRH (approximately 2.5 mg/ml) and the mixture was incubated at 37°C for 1 hour. Following the incubation the sample was applied to a Ultramax 5,000 MW cut-off centrifuge filter to remove the protein. To assess for AI-2 activity 10 µL of the filtrate was used in the *Vibrio harveyi* AI-2 bioassay as described above. When the sample was to be used for quantification of homocysteine formation the incubation was modified slightly. For these experiments 50 µL of recombinant enzyme (1 mg/mL) was added to 250 µL of SRH (∼2.5 mg/mL) and the incubation time was shortened to 15 minutes due to the instability of the homocysteine at 37°C [37].

#### Measurement of homocysteine formation by recombinant LuxS proteins

In order to measure the homocysteine formation by the recombinant LuxS proteins a standard Ellman reaction was utilized to measure the formation of free thiol groups as previously described [37]. Briefly, an equal volume of the DTNB (Sigma) working stock (5 mM) was added to the filtrate obtained following the synthesis reaction. The absorbance at 412 nm was used to quantitate the homocysteine formed by the reaction.

#### LuxS Activity Assay

LuxS reactions were performed in a buffer containing 50 mM HEPES (pH 7.5), 150 mM NaCl, 150 µM 5,5′-dithio-bis-(2-nitrobenzoic acid) (DTNB) and various concentrations of SRH (0–80 µM). The reactions were initiated by the addition of LuxS (final concentration 1.3 µM for wild-type and 8.4 µM for G92D mutant) and monitored continuously at 412 nm (ε = 14150 M^−1^ cm^−1^) in a Perkin-Elmer λ25 UV-VIS spectrophotometer at room temperature (23°C). The initial rates recorded from the early regions of the progress curves were fitted against the Michaelis-Menten equation *V*  =  *k*_cat_ [E]_0_ [S]/(*K*_M_ + [S]) using KaleidaGraph 3.5 to determine the kinetic constants.

#### Quantitation of AI-2 in Culture Fluids

Cell free supernatants of both 81116AI2- and 81116AI2+ cells were collected at specific time points during a 24-hour growth period. The cell density was determined by measuring the OD_600_. The cultures were centrifuged and filtered through a 0.25 µM Acrodisc filter to remove bacterial cells. The resulting supernatant (30−60 µL) was added to 1.0 µM LuxP137Dap in 50 mM Hepes (pH 7.0) and 150 mM NaCl, and the fluorescence spectra were recorded before and after incubation with 1.6 mM borate for 5 min at room temperature (total volume 1 ml) [39]. The AI-2 concentration was calculated from the fluorescence increase at 494 nm using known concentrations of DPD as calibration standards.

## Results

### Identification of a naturally occurring AI-2 deficient variant of *C. jejuni*

**Figure 1:**
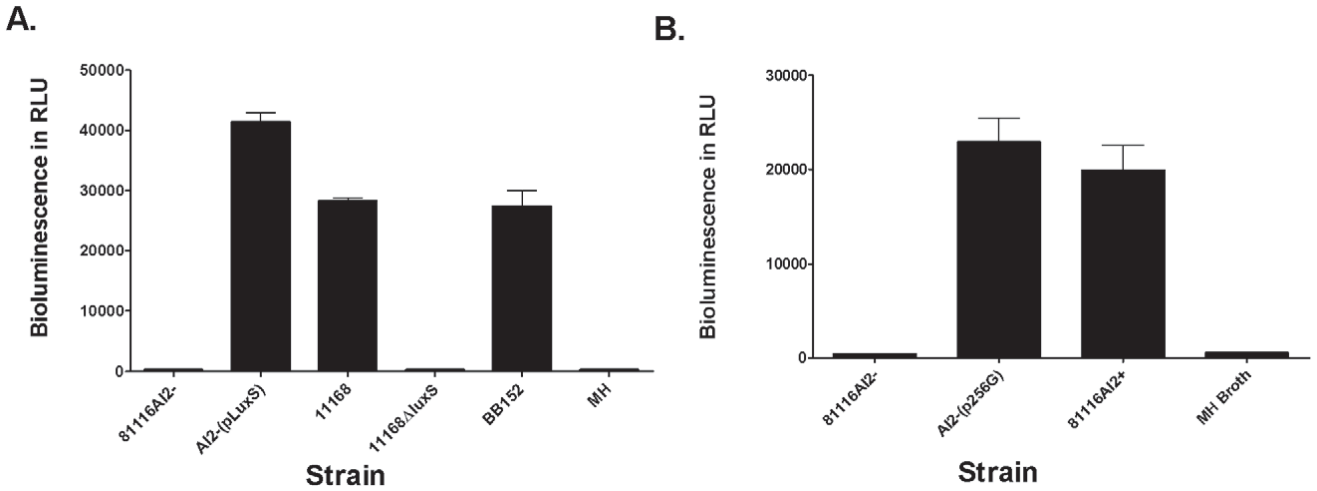
Autoinducer-2 activity of strains used in study. A: Bioluminescence activity of 81116AI2- and AI2-(pLuxS) compared to the positive control strains *Campylobacter jejuni* 11168 and *Vibrio harveyi* BB152. Each bar represents the average of three replicates with standard deviation. MH both is shown as a negative control. BB152 – positive control B: AI-2 production of 81116 variants and conjugates. 81116AI2-, a laboratory strain with the G92D mutation in LuxS (AI-2 negative), AI2-(p256G), a 81116 conjugate with a shuttle vector carrying a *luxS* gene in which the G92D mutation is reverted; 81116AI2+, a 81116 variant with no mutation in *luxS* (AI-2 positive); and MH Broth, culture media serving as a negative control for AI-2 activity.

**Figure 2:**
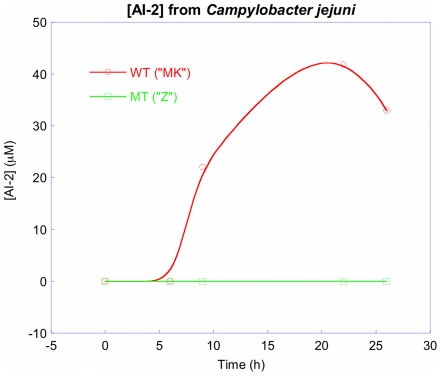
AI-2 concentration curves for the two recombinant proteins as a function of time and measured by FRET.

Several *C. jejuni* isolates commonly used for laboratory research were screened for their ability to synthesize AI-2. Surprisingly, the 81116 strain maintained in our laboratory (named 81116AI2- in this study) was found to be deficient in AI-2 synthesis when assessed by the *V. harvyei* AI-2 bioassay and compared to the bioluminescence obtained with other *C. jejuni* isolates and the negative controls. Unlike the positive control strains, 81116AI2- did not produce AI-2 during any stage of the growth phase. (Figure 1A) Since the *V. harveyi* bioassay is not quantitative, we next determined the concentration of AI-2 in the culture fluids of 81116AI2+ and 81116AI2- cells using a LuxP-based fluorescent sensor recently developed in one of our laboratories [39]. For this experiment, 81116AI2+ and 81116AI2- were grown in MH media under identical conditions. A sample of the culture media was collected at specified time points and the cell free supernatant was collected and mixed with the LuxP137Dap sensor protein (in the presence of 1.6 mM borate), and the fluorescence yield at 494 nm was measured. Comparison with AI-2 standards confirmed that the 81116AI2+ cells produced significant levels of AI-2 starting at ∼9 h and the AI-2 level peaked at 42 µM at ∼22 h (Figure 2). In contrast, the 81116AI2- cells produced no detectable amounts of AI-2 (detection limit ∼1 µM) at any stage. Complementation of the strain with a plasmid encoded copy of the *luxS* ORF from an AI-2 producing strain of *C. jejuni* (NCTC 11168) resulted in the production of AI-2 at levels comparable to positive control strains (Figure 1A). Since strain 81116 was know to be positive in previous studies by other laboratories [8], we obtained another copy (named 81116AI2+ in this study) of strain 81116 from an independent laboratory (Michael Konkel, Washington State University) to verify the AI-2 deficient phenotype observed in strain 81116AI2-. It was found that the AI-2 level of 81116AI2+ as measured by bioluminescence assay was essentially identical to those produced by NCTC11168 and the VhBB152 positive control strain (Figure 1B). These findings suggest that 81116AI2- is an AI-2 deficient variant of strain 81116 and may harbor a genetic change that is responsible for the loss of AI-2 production.

### A single point mutation in LuxS is responsible for the loss of AI-2

**Figure 3:**
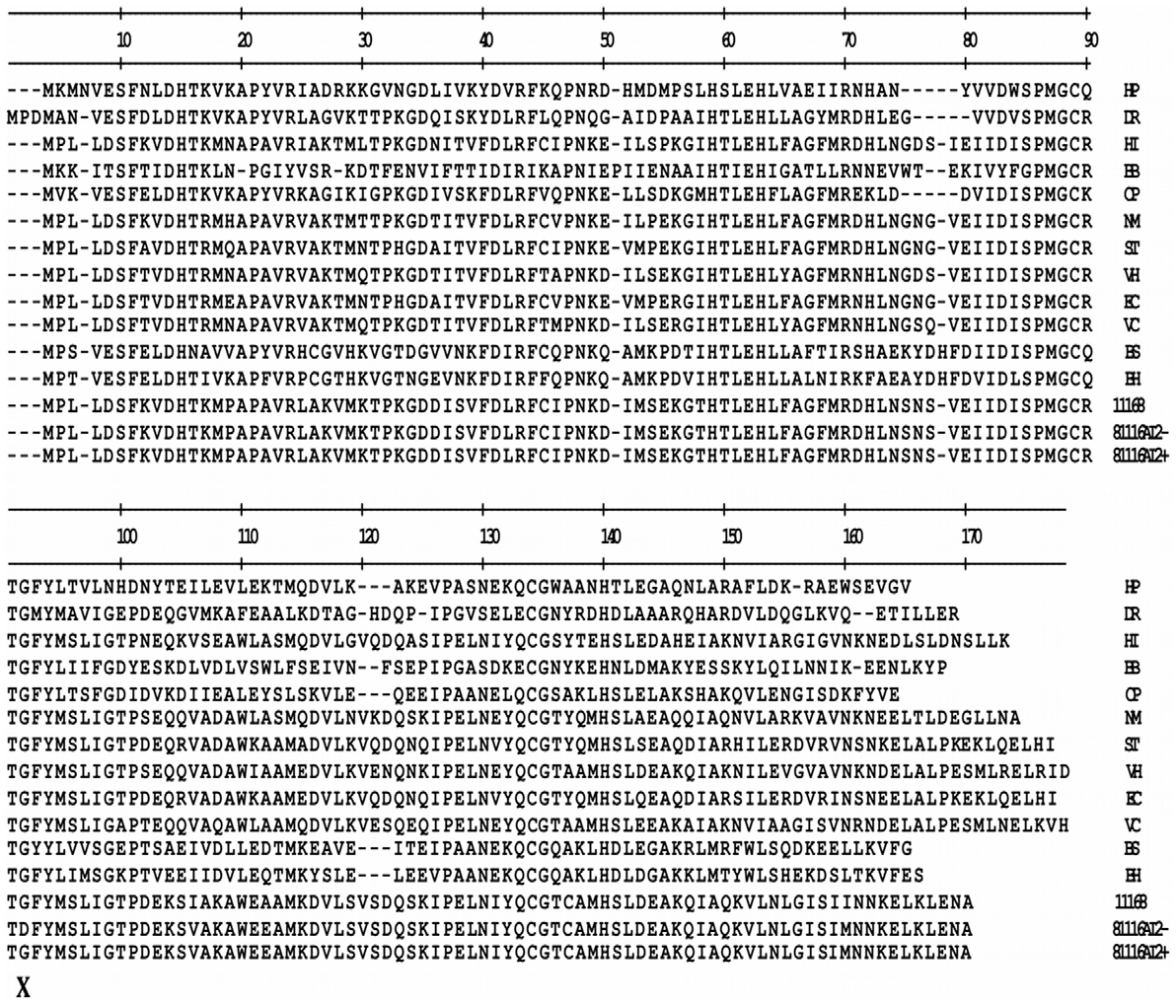
Sequence alignment of *luxS* genes including 81116. Highlighted amino acids are mutated in the 81116 (bottom row) compared to the published 11168 sequence (2^nd^ from bottom). “**X**” denotes the site of amino acid substitutions. Proteins used in the sequence alignment are as follows (Protein Information Resource database ID numbers are shown in parentheses, with GenBank ID numbers given for ST, VH, and BH): HP, *Helicobacter pylori* (C71973); DR, *Deinococcus radiodurans* (D75280); HI, *Haemophilus influenzae* (G64008); BB, *Borrelia burgdorferi* (BB0377); CP, *Clostridium perfringens* (T43793); NM, *Neisseria meningitides* (G81963); ST, *Salmonella typhimurium* (AAF73475.1); VH, *Vibrio harveyi* (AAD17292.1); EC, *Escherichia coli* (H65048); VC, *Vibrio cholerae* (F82309); BS, *Bacillus subtillis* (A69994); and BH, *Bacillus halodurans* (BAB07072.1).11168, *Campylobacter jejuni* ATCC 11168; 81116AI2-, *Campylobacter jejuni* Strain 81116 from this study; 81116AI2+, *Campylobacter jejuni* Strain 81116 from this study.

To determine the genetic basis for the phenotypic difference between 81116AI2- and 81116AI2+, we compared the sequences of the *luxS* gene in the two variants. No difference was observed in the promoter region of the *luxS* gene, however, a single adenosine to guanosine mutation was observed at position 256 of the *luxS* gene in 81116AI2-. The mutation results in the substitution of an aspartic acid for glycine-92 in the LuxS protein. Sequence alignment of 14 LuxS proteins from a wide range of bacterial species revealed that the glycine residue is conserved across all species (Figure 3). To determine whether the G92D mutation is responsible for the loss of AI-2 production, we mutated the Asp-92 back to a glycine by site-directed mutagenesis. Introduction of a plasmid copy of the revertant *luxS* gene into the 81116AI2- strain restored its ability to produce AI-2 to the same level as the 81116AI2+ strain (Figure 1B). Thus, the deficiency in AI-2 production is caused by the G92D mutation.

### The G92D mutant LuxS protein is catalytically deficient

**Figure 4:**
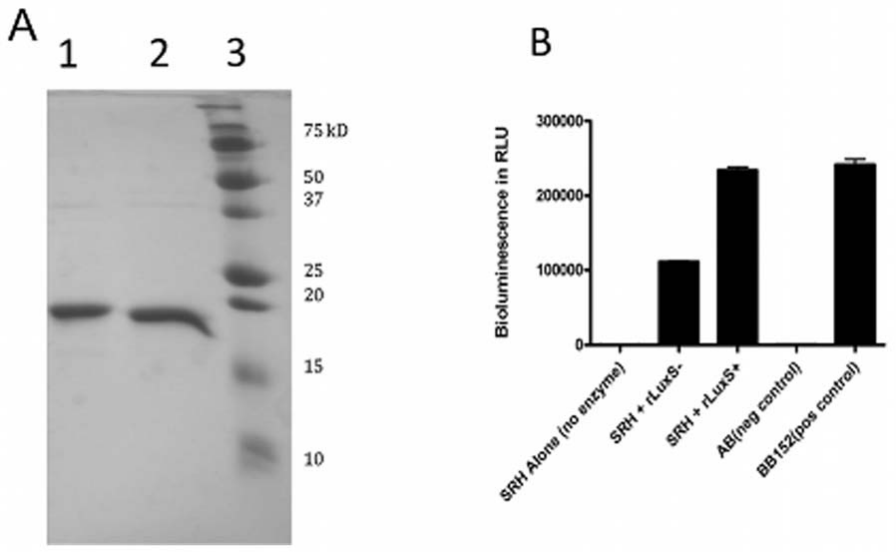
The purity and activity of recombinant LuxS proteins synthesized in this paper. A: SDS-PAGE gel of recombinant LuxS proteins from 81116AI2- and 81116AI2+ Lane 1: rLuxS- (expected MW = 18.1 kD), the recombinant 81116AI2- 6HIS-tagged LuxS Protein, Lane 2: rLuxS+ (expected MW = 18.1 kD), the 81116AI2+ 6HIS-tagged LuxS Protein, Molecular weight markers as shown. B: Enzymatic activity of recombinant LuxS protein measured by *Vibrio harveyi* bioluminescence assay. SRH alone with no enzyme provides a negative control. BB152 is cell-free supernatent of *V. harveyi* BB152 as a positive control.

**Figure 5:**
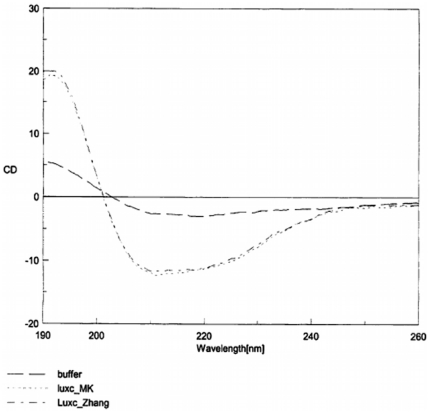
Circular dichroism spectra of the rLuxS+ and rLuxS- recombinant proteins. The two protein spectra essentially overlay each other identically throughout the spectra. The buffer is also shown as a control.

**Figure 6:**
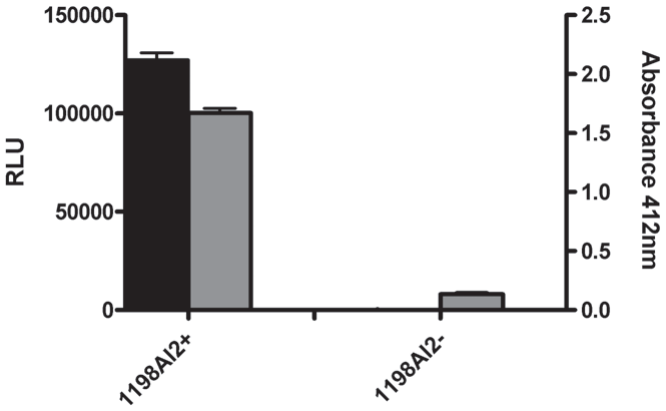
Recombinant protein activity of the AI2^+^ and AI2^-^ strains used in this paper. A. Paired homocysteine and AI-2 data from the LuxS recombinant proteins. rLuxS+ is the recombinant LuxS protein from 81116AI2+ and rLuxS- is the recombinant protein from 81116AI2-. Black bars represent the homocysteine levels measured using the DNTB reaction measured by Absorbance at 412 nm (right axis). The grey bars represent the AI-2 values measured by V. harveyi bioluminescence (left axis). Values for each sample were measured on split samples.

zTo determine whether the G92D mutant LuxS is catalytically deficient, we overexpressed both the wild-type and mutant LuxS proteins in *E. coli* with an N-terminal six-histidine tag and purified the recombinant proteins to near homogeneity by metal affinity chromatography (Figure 4A). The circular dichroism spectra of the two proteins are identical, suggesting that the mutation does not significantly alter the secondary structure of the protein (Figure 5). Kinetic analysis showed that the wild-type LuxS enzyme from 81116AI2+ had a *k*_cat_ value of 0.009 s^−1^, a *K*_M_ value of 28 µM, and a *k*_cat_/*K*_M_ of 332 M^−1^ s^−1^. In contrast, the activity of the 81116AI2- (G92D) LuxS enzyme was to low to allow for accurate measurement of its *k*_cat_ and *K*_M_ values. Its *k*_cat_/*K*_M_ value was estimated to be ∼3.5 M^−1^ s^−1^, which is a 100-fold lower than that of the wild-type enzyme. Therefore, while the G92D mutant retains residual catalytic activity in vitro (figure 6), this 100-fold reduction in activity results in a phenotypic loss of AI-2 production in *vivo*.

### Evaluation of SRH levels in the cell free supernatant of cultures

**Figure 7:**
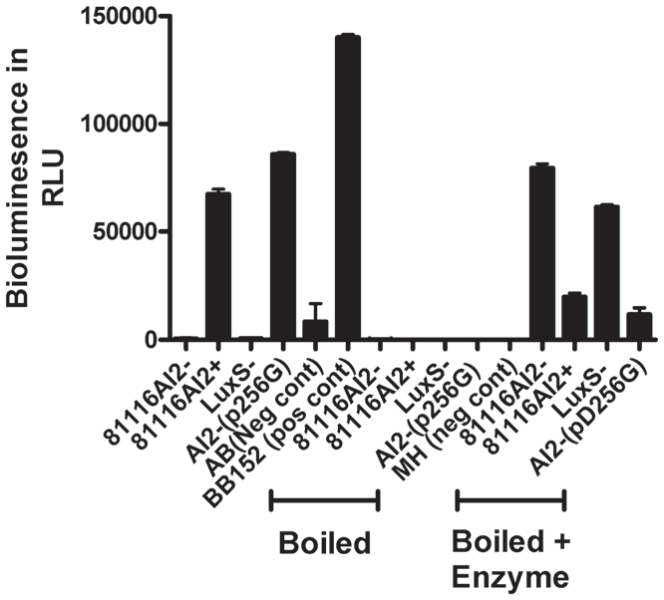
AI-2 and SRH levels of various cultures. The first 6 columns depict the cell-free supernatent AI-2 concentrations of cultures as a reference for endogenous LuxS activity. The four columns labeled boiled are cell-free supernatent post-boiling which destroys AI-2 activity. Finally the last 4 columns show the AI-2 activity produced in the presence of exogenous rLuxS+ recombinant protein which provides a semi-quantitative measure of extracellular SRH levels.

Given that the LuxS protein of 81116AI2- is significantly reduced in its ability to convert SRH to homocysteine and DPD, we were interested in testing the SRH levels in the cell free media of the various bacterial cultures. Previous work in other species has demonstrated elevated SRH concentrations in the culture supernatant of *luxS* mutant strains [40]. To test this we utilized the *in vitro* AI-2 synthesis reaction described above with the wildtype LuxS enzyme and the cell-free supernatant as the source of substrate. In order to prevent any cross contamination of the reaction with endogenously produced AI-2 the supernatant was boiled for 5 minutes prior to the reaction then allowed to cool. The data presented in figure 7 demonstrates that strains deficient in their ability to produce AI-2 (81116AI2- and LuxS-) yield higher RLU following *in vitro* synthesis when compared with AI-2 competent strains [81116AI2+ and AI2-(pD256G)]. Elevated RLU units following the reaction are consistent with the presence of an increased SRH concentration in the supernatant that is reacted to produce AI-2 in the presence of the functional recombinant LuxS enzyme.

## Discussion

*C. jejuni* 81116 is a commonly used laboratory strain that was originally isolated from an outbreak of enteritis in a UK boarding school in 1983 [31]. Since that time the strain has been widely disseminated and maintained in laboratories around the world. Despite the fact that this strain of *C. jejuni* is laboratory adapted and has been subjected to repeated laboratory passage, evidence suggest that the isolate has maintained an amazing level of genetic stability over the last 20 years and that the AFLP pattern determined in 2001 was identical to the co-isolates that had not been passaged since 1983 [41]. Despite this considerable genome stability, the data presented herein clearly demonstrates the presence of a single nucleotide substitution in the open reading frame of the *luxS* gene of 81116AI2- that results in a single amino acid substitution and the loss of AI-2 phenotype. Identification of such a mutation is not precluded by the genetic stability described above since the sensitivity of the AFLP technique for the detection of specific single amino acid changes is extremely low for the portions of the genome not included in the restriction enzyme recognition sites.

Interestingly, two previous studies regarding AI-2 competence of *C. jejuni* have utilized isolates of 81116 and have demonstrated it to be AI-2 positive [8], [30]. To confirm our results we were able to obtain genomic DNA of the 81116 isolate used by one of those laboratories as well as a second 81116 isolate (81116AI2+) originating from a third laboratory. Our results indicated that 81116AI2- is a variant of strain 81116 and harbors a single nucleotide substitution in *luxS.* This mutation results in the substitution of an invariant glycine residue for a much larger charged aspartate residue in LuxS. Based on crystallographic structures of several LuxS enzymes this residue is located at the amino end of β-strand 4 [42]. In this position it is located within a fraction of an angstrom from both the Cis-84 residue located 2 residues upstream and the Arg-39 residue located on the adjacent β2-strand. Both of these amino acids play important roles in the kinetics of the enzymatic reaction and the stabilization of the enzymatic intermediates [14], [17]. We speculate that the substitution with a larger charged amino acid induces steric hindrance and electrostatic repulsion, which induces conformational changes that result in decreased enzymatic kinetics. Specifically, we speculate that the presence of the larger aspartate residue results in either the β4 strand being displaced away from the active site and pulling the Cis-84 residue out of its typical 3 dimensional position or the displacement of the β2 strand towards the active site, resulting in the Arg-39 residue displacement. In either case the relative locations of these two residues would be disrupted, negatively impacting enzymatic function. Given the lack of significant change in circular dichroism spectra and the retention of some enzymatic activity, this conformation change is assumed to be minor and may only result in slight repositioning of some key active site residues.

To our knowledge, while at least one other naturally occurring *E. coli luxS* mutant has been described [43], this isolate represents the first description of a naturally occurring *luxS* mutant phenotype in *C. jejuni*. Previous studies have utilized site directed mutagenesis to explore the amino acids involved in the active site and have demonstrated significant reductions in the enzyme activity associated with mutagenesis of these active site residues [14], [17]. Data herein demonstrated that the single amino acid substitution described in this isolate results in significant reductions in enzymatic activity and ultimately the loss of AI-2 phenotype. The circular dichroism spectra of the wildtype and mutant LuxS were nearly identical (figure 5), suggesting that the secondary structures of the proteins are largely conserved. While the ability of circular dichroism to accurately predict secondary structure *in silico* is limited, its ability to compare two similar proteins secondary structure is much more robust [44].

Results of both the *V. harveyi* bioluminescence and FRET method demonstrate the phenotypic loss of AI-2 in the 81116AI2- variant when compared to 81116AI2+ and other *C. jejuni* strains (figures 1B and 2). In contrast, the *in vitro s*-ribosylhomocysteinase enzymatic kinetics demonstrated that relatively low levels of enzymatic activity were still retained in the mutant LuxS. This retained but limited enzymatic ability explains the detection of AI-2 production following *in vitro* incubation of concentrated recombinant mutant enzyme with excess of SRH (figures 4 and 6). Based on the enzyme kinetics of the wildtype protein kcat/Km of 332/M/s and that of the mutant protein at roughly 3.5/M/s we can estimate that the mutant protein has a roughly 100-fold decrease in enzymatic activity. As a result, we believe that the physiological concentrations of AI-2 produced during the *in vitro* growth of 81116AI2-, using the media described, fall below the limits of detection of our AI-2 assays (LOD for FRET ∼1 uM) resulting in a phenotypic loss of AI-2 activity while maintaining a minimally-functional LuxS enzyme. Such levels would also fall well below the 40 uM concentrations observed in the wildtype strain during growth in the same media, however data regarding the concentration of AI-2 necessary to result in signaling of *C. jejuni* is currently unavailable and thus interpretation of the biological meaning of these levels in hampered. The biological role of AI-2 competent strains of *C. jejuni* is still unclear. Evidence suggest that *luxS* mutants have both altered phenotypes and altered gene expression, however some of these changes are clearly associated with altered metabolism and the role of AI-2 mediated quorum sensing in this species remains uncertain [45], [46]. Furthermore, *luxS* mutants do demonstrate an altered ability to colonize animal models [47]. In the present study the mutant exhibited a decreased motility consistent with previously published findings of *luxS* mutants in this species [8], [30].

The use of the FRET AI-2 assay in this manuscript provides, to our knowledge, the first truly quantifiable data concerning the physiological concentrations of AI-2 produced by *C. jejuni* grown in *in vitro* environments. While the ability of the organism to truly “sense” the compound is still unproven, this quantification data does provide much needed information concerning what range of potential concentrations of chemically synthesized AI-2 should be used to mimic natural production potentials. Based on our results we believe that concentrations between 0 and 40 uM are physiologically possible in logarithmic growth in MH media routinely used for *C. jejuni* growth.

When comparing the results of the *in vitro* recombinant proteins enzymatic reactions we observe that while no homocysteine can be detected following incubation of SRH with the 1198AI2- enzyme we do see measurable synthesis of AI-2 (figure 6). This provides additional evidence of retention of low-level enzymatic activity of the 81116AI2- strain but also provides clues into the biological sensitivity of two of the assays utilized in detection of LuxS enzymatic activity, homocysteine formation using the Ellman reaction and AI-2 activity using the *Vibrio harveyi* assay. Given that homocysteine will be produced in the *in vitro* reaction in a stoichiometrically identical concentration to DPD, that then becomes AI-2, the ability to detect low levels of AI-2 in the absence of homocysteine suggest that the lower limit of detection of the Ellman reaction, as performed in this manuscript, is greater then that of the *Vibrio* bioluminescence assay.

As has been described for other organisms, the absence of a fully functional *luxS* gene in *C. jejuni* does result in increased extracellular concentrations of SRH (Fig. 7). The presence of increased SRH concentrations in the naturally occurring mutant strain suggest that despite measurable residual enzymatic activity of the mutant enzyme, the metabolic ability of the organism to recycle SRH into the SAM recycling pathway is hindered. Thus, in addition to the phenotypic loss of AI-2 activity this strain has a phenotypic change in SAM recycling which precludes the ability to attribute any of the phenotypic characteristics observed directly to the loss of quorum sensing.

In conclusion, we have fully described the molecular basis for the loss of AI-2 phenotype associated with a naturally occur *luxS* mutant of *C. jejuni*. Given that the amino acid responsible for this loss of function is highly conserved over a broad range of bacterial species, we speculate that homologous mutations in other bacterial species would also result in a loss of AI-2 phenotype. Additionally, the fact that this amino acid mutation allows for the maintenance of relatively normal secondary structure while diminishing enzymatic activity to levels similar to that observed with *luxS* deletion mutants suggest that this amino acid is critical for normal enzyme function. Use of this isolate may be helpful in further defining the roles of AI-2 in quorum sensing in a wide variety of bacterial species since it does provide a potential enzymatic intermediate between fully functional and enzymatically incompetent.
